# Ultraviolet Technologies for Yeast Control and Functional Modulation in the Food Industry: Mechanisms, Resistance and Applications

**DOI:** 10.3390/foods15061102

**Published:** 2026-03-21

**Authors:** Agustín Zavala, Oscar Cavieres, Mariela Labbé, Fernando Salazar

**Affiliations:** Laboratorio de Fermentaciones Industriales (IFELAB), Escuela de Alimentos, Facultad de Ciencias Agronómicas y de los Alimentos, Pontificia Universidad Católica de Valparaíso, Av. Waddington 716, Valparaíso 2360100, Chile; agustin.zavala@pucv.cl (A.Z.); oscar.cavieres@pucv.cl (O.C.); mariela.labbe@pucv.cl (M.L.)

**Keywords:** UV-LED, UV-C irradiation, yeasts, *Saccharomyces cerevisiae*, microbial inactivation, DNA damage, photoreactivation, mutagenesis, food fermentation

## Abstract

Yeasts play a vital role in food fermentation processes, where their viability, stress tolerance, and metabolic performance directly influence product quality and process efficiency. Controlling and modulating yeast behavior represents a challenge in the food industry, particularly in non-thermal processing contexts. Ultraviolet (UV) technology has traditionally been applied as a microbial control tool; however, yeast response mechanisms to UV irradiation extend beyond simple inactivation. Depending on wavelength, dose, and treatment conditions, UV exposure can lead to complete inactivation, partial reduction in viability, or induce stable phenotypic changes associated with cellular stress responses and Deoxyribonucleic Acid (DNA) damage processing. This review examines current knowledge on yeast–UV interactions across different food matrices, highlighting how UV treatments influence yeast physiology and functionality. In addition, recent studies suggest that UV-induced genetic alterations, when properly controlled, may contribute to yeast diversification and functional modulation without the use of genetically modified organisms. The review discusses technological opportunities, practical limitations, and future research needs, emphasizing the dual role of UV technology as a tool for yeast control and as a potential driver of functional modulation.

## 1. Introduction

Yeasts play a dual and fundamental role in the food industry due to their metabolic versatility and taxonomic diversity. While *Saccharomyces cerevisiae* remains the most extensively exploited yeast species in industrial fermentations, non-*Saccharomyces* yeasts have gained increasing recognition for their complex fermentation pathways, aroma modulation, and product differentiation [1]. However, this same physiological variability, which includes differences in metabolic capacity, stress tolerance, and adaptation, also makes yeasts significant spoilage agents. Their ability to remain active under acidic conditions and high sugar concentrations can lead to quality losses such as turbidity and off flavors [1,2,3]. This heterogeneity implies that the biological features enabling technological exploitation can also hinder microbiological control [1,4]. Consequently, there is a critical need for preservation strategies, such as ultraviolet radiation, that account for differences in susceptibility rather than applying uniform inactivation approaches across heterogeneous yeast populations, enabling not only microbial control but also selective modulation of yeast populations [3,5,6].

Ultraviolet (UV) radiation is an emerging tool in the disinfection and microbiological control of food and beverages in the food industry. It is notable for its non-thermal nature, low environmental impact, and ability to preserve the sensory and nutritional quality of treated products [6,7]. In particular, technology based on ultraviolet light-emitting diodes (UV-LED) has gained increasing interest in the food industry due to its energy efficiency, long service life, and ability to operate at specific wavelengths, such as UV-A (315–400 nm), UV-B (280–315 nm), UV-C (200–280 nm), and vacuum UV (VUV) (100–180 nm) [8,9]. The effect of each wavelength range on microorganisms varies, making it an emerging technology in the field of food decontamination.

**Figure 1:**
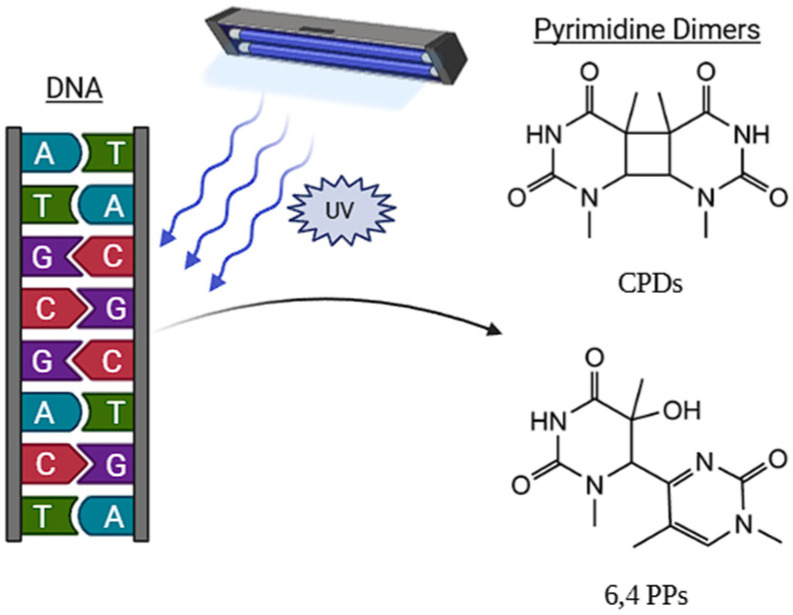
Dimerization of pyrimidine bases induced by UV light and formation of cyclobutane pyrimidine dimers (CPDs) and pyrimidine 6-4 pyrimidone (6-4 PPs) products.

The antimicrobial action of UV radiation is divided into two main mechanisms: direct interaction with DNA nucleic acids and the generation of reactive oxygen species (ROS) [10,11]. UV radiation in the form of photons penetrates genetic material until it interacts with nitrogenous bases, pyrimidines and purines, transferring energy, electronically exciting them, and forming photoproducts (Figure 1) [10,11,12]. These irreversible covalent bonds distort the DNA structure, interfering with replication and transcription, generating mutations that can result in the loss of cell viability [13,14]. Similarly, UV radiation can induce the formation of ROS, which causes oxidative damage to proteins, lipids, and nucleic acids, affecting the integrity and functionality of the cell wall of microorganisms [15,16]. In yeasts, however, the presence of β-glucans and mannoproteins in species such as *Saccharomyces cerevisiae*, contributes to mechanical stability and may limit ROS diffusion. In addition, several yeasts, such as *Rhodotorula* (e.g., *R. mucilaginosa* and *R. glacialis*), synthesize photoprotective compounds and accumulate carotenoids, including torularhodin, which enhance UV tolerance through antioxidant activity and energy dissipation [17]. Likewise, mycosporine production has been reported in genera such as *Dioszegia*, *Cryptococcus*, and *Pichia*, where these UV-absorbing molecules (310–320 nm) contribute to photoprotection and oxidative stress mitigation [18,19].

The susceptibility of microorganisms to damage caused by UV light varies significantly between species and strains, due to structural and physiological factors such as cell wall composition, cell size, conformation, and DNA pyrimidine content [20,21]. In general, the greatest germicidal effect occurs in the UV-C classification. Ref. [22] describes that this effect in bacteria varies depending on whether they are Gram-positive or Gram-negative, due to the characteristics of their cell walls. In the case of yeasts, their greater resistance to UV-C light irradiation is associated with their larger cell size (approximately 8–10 μm in diameter), which is greater than that of typical bacterial cells such as *E. coli* and *L. innocua* (generally 0.5–0.3 μm in diameter), as well as with the presence of DNA repair mechanisms, including photoreactivation (PR), nucleotide excision repair (NER), and recombinational (or post-replicative) repair (HR) which reduces the effectiveness of the treatment to inactivate them [23,24].

This review critically analyzes bibliographic information on the effect of UV irradiation on yeasts, analyzing the structural, physiological, and molecular factors that determine their susceptibility, as well as the technological consequences of this emerging technology.

## 2. Yeasts in the Food Industry

In the food industry, yeasts are a diverse group of species commonly found in food matrices at different stages of production, processing, and storage, often reflecting the physicochemical characteristics of the substrate and the surrounding microbial ecosystem. In fermented foods and beverages, yeasts are primarily introduced intentionally as starter cultures or arise naturally from raw materials, where their ability to utilize fermentable sugars and withstand fermentation conditions allows them to prevail [25,26]. On the other hand, in non-fermented or minimally processed foods, yeast presence is typically incidental and linked to environmental contamination, growth on foods during postharvest handling and storage, or processing conditions that favor yeast survival over bacteria [27].

In this context, different groups of yeasts are commonly associated with specific food matrices. For example, *Saccharomyces* species dominate alcoholic fermentations, while species such as *Candida*, *Pichia*, *Metschnikowia*, *Rhodotorula*, *Aureobasidium*, and *Cryptococcus* are frequently reported in fruit-derived products and postharvest environments, where they may act either as spoilage organisms or as antagonists against plant pathogens [27,28,29]. This matrix-dependent distribution highlights that yeast functionality cannot be generalized across food systems, as the same yeasts may behave differently depending on substrate composition, competing microorganisms, and processing goals [25].

Yeasts may also be present in foods indirectly, through the use of yeast-derived ingredients and compounds. Yeast cells have been extensively explored as sources of proteins, vitamins, minerals, and structural polysaccharides, positioning them as technologically relevant ingredients in the food industry [30,31]. At the same time, specific yeast species have demonstrated antagonistic activity against foodborne pathogens, highlighting that yeasts can influence microbial kinetics in foods through competition and inhibition [32]. These observations show that the diversity of yeast species strongly depends on the food matrix and processing parameters, which should be considered when evaluating preservation strategies.

## 3. UV-Based Technologies: Characteristics and Processing Parameters

### 3.1. Low-Pressure Lamps (LP-UV)

Low-pressure lamps (LP-UV) have been the dominant technology and industry standard for UV disinfection for the last few decades [33,34,35]. Their operating principle is based on the emission of an electric discharge, which generates an electric arc to ionize an inert gas (usually argon or xenon). This vapor excites the mercury or metal amalgam (mercury/gallium or mercury/indium) contained inside a glass or quartz tube, emitting photons in the monochromatic UV light range [36]. Approximately 85% of its energy is concentrated at a single wavelength of 253.7 nm, which is characterized by its germicidal potential [37]. The maturity of this technology makes it highly reliable, with low manufacturing costs and high power output, which explains its economic appeal and widespread adoption in the medical, water treatment, and food industries.

However, LP-UV lamps have drawbacks that should not be ignored. The main one is that they operate using mercury, which is a powerful neurotoxin that poses a critical contamination risk, on breakage, especially in sensitive environments such as the food industry [38,39]. On the other hand, prolonged exposure to UV-C light can cause erythema and photokeratitis [40]. In addition, regarding the operation disadvantages, LP-UV lamps need a preheating time to reach their optimal radiance, with limited lifespan (8000–12,000 h) and their fragility, being manufactured with glass or quartz increases the risk of mechanical failure.

### 3.2. UV Light-Emitting Diodes (UV-LED)

UV light-emitting diodes (UV-LEDs) represent an emerging technology in the food industry as they are non-thermal, leave no chemical residues during the process, and are non-toxic, directly addressing the shortcomings of LP-UV lamps [41]. Their most notable advantage is that they are completely mercury-free, positioning them as the leading safe and environmentally friendly alternative, completely eliminating the risk of mercury contamination in the event of damage [42,43].

Among their operational advantages, unlike conventional lamps that require heating, UV-LEDs have the ability to operate at full intensity instantly, making them ideal for integration into automated production lines [44,45]. Their useful life exceeds 30,000 h, drastically reducing maintenance costs and downtime. Furthermore, the nature of this technology allows for wavelength selectivity, enabling the manufacture of LEDs with specific wavelengths, facilitating the optimization of the inactivation process for specific microorganisms, since the highest absorption of photons in DNA is around 265 nm [46].

The transition from LP-UV lamps to LED technology represents a paradigm shift in the design of equipment incorporating UV systems. On one hand, LP-UV lamps can reach lengths of up to 16 cm, whereas LEDs have an approximate emitting area of only 1–4 mm^2^. In addition, LP-UV lamps emit energy as UV radiation and thermal energy in an omnidirectional manner (360°), while LEDs release heat in the direction opposite to where the UV light is emitted. This characteristic enables the development of LED-based systems that can be adapted to complex geometries, whether integrated into a processing line or within a closed bioreactor system [47]. Consequently, this technology represents a significant shift in the design of reactors equipped with UV decontamination systems [44,48].

### 3.3. Pulsed UV Light

Pulsed UV light (PL-UV) represents a distinct technological approach for disinfection [49]. It uses xenon gas lamps to emit pulses of extremely high-intensity light [50,51] with extremely short durations, on the order of microseconds [52,53]. Unlike continuous UV sources, pulsed light is broad-spectrum (polychromatic), emitting across a very wide range from UV (typically around 200 nm) to the near-infrared region (1100 nm) [49,51,54]. This characteristic underlies its powerful and unique mechanism of action.

The germicidal power of pulsed light is not based solely on photochemical damage to DNA, as is the case with conventional UV-C [49]. Instead, it employs a synergistic inactivation mechanism that combines multiple effects [54]. First, the UV component (primarily UV-C) induces DNA damage through dimer formation [51,52]. However, pulsed UV light also exhibits a threshold effect in which microbial lethality increases once the pulse intensity is sufficient to induce structural damage. Yeast exposed to flashes of UV doses (0.23 J cm^−2^) but different peak powers (2473 and 4655 kW) showed markedly greater protein leakage and membrane disruption at higher peak power [55]. In addition, 6-log reductions have been achieved at total fluences between 2.16 and 3.94 J cm^−2^, depending on spectral composition and pulse characteristics [56]. Similarly, PL-UV treatments around 0.8 J cm^−2^ have produced 5-log yeast reductions accompanied by morphological alterations consistent with membrane destabilization [57]. These findings indicate that microbial inactivation with PL-UV depends not only on total fluence but also on reaching pulse conditions that promote photophysical and photothermal membrane damage in addition to DNA photochemistry [46,52,55,58].

This combination of effects makes PL-UV extremely effective, surpassing cellular photorepair mechanisms by causing irreversible damage to proteins and membranes, and demonstrating high lethality even against highly resistant microorganisms such as bacterial and fungal spores [46,52]. Due to its low penetration in opaque matrices and its high intensity, its primary application is the decontamination of solid food surfaces, such as meats, fish, fruits, and packaging materials [49,53,59,60]. It is important to note that although PL-UV is often classified as a non-thermal technology, the inclusion of a strong photothermal component implies that the surface temperature of the food may increase, which must be considered for products sensitive to such temperature changes [51].

## 4. Effects of UV Radiation on Yeasts

### 4.1. Importance and Intrinsic Resistance of Yeasts

The implementation of non-thermal technologies, such as UV radiation, has gained significant interest as an alternative to traditional thermal pasteurization [61,62]. The objective is to control pathogenic and spoilage microorganisms in foods and beverages while minimizing the negative impact on nutritional and sensory quality often caused by thermal treatments [37]. In the context of fruit juices and acidic beverages, yeasts represent a particular microbiological challenge. These microorganisms are the primary agents responsible for spoilage in such products, capable of causing undesired fermentation, turbidity, gas production, and off flavors [63,64]. Species of the genus *Zygosaccharomyces* are notorious for their high tolerance to multiple stress conditions, such as low pH and high sugar concentrations [63,65,66]. In the wine industry, species like *Brettanomyces bruxellensis* are feared due to their ability to produce volatile phenols, which impart undesirable aromas to wine [54,67,68]

A key challenge in the implementation of UV technology is that yeasts, in general, display significantly higher resistance to UV-C radiation compared to bacteria [62,69,70]. This intrinsic resistance is attributed to several factors. First, the considerably larger cell size of yeasts (eukaryotes) increases the frequency which UV photons are absorbed or scattered by other cellular components before reaching and damaging the nuclear genetic material [62,71,72]. Yeasts possess a complex cell wall and a nucleus that houses the genetic material, providing a physical and structural barrier not present in prokaryotic bacteria [62]. Genomic base composition has also been proposed as a factor influencing UV-C susceptibility, given that photochemical damage primarily occurs through the formation of cyclobutane pyrimidine dimers and 6–4 photoproducts at adjacent pyrimidine sites. In the applied UV literature, yeasts have occasionally been described as potentially less susceptible due to differences in nucleotide composition [70]. However, genomic Guanine–Citosine (GC) content varies substantially across microorganisms, particularly among bacteria, where it ranges approximately from 17% to 75% [73,74]. In comparison, *Saccharomyces cerevisiae* exhibits a genomic GC content of approximately 38% [75]. Therefore, rather than assuming a universally lower pyrimidine content in yeasts, a more precise interpretation is that organism-specific GC and Adenine-Thymine (AT) composition may influence the density of UV-reactive dipyrimidine sites, and this parameter should be evaluated at the genome or strain level rather than generalized across domains.

### 4.2. Inactivation Mechanisms and Variability Among Species

The primary inactivation mechanism of UV-C radiation (200–280 nm), whether from LP-UV or UV-LED systems, is the direct photochemical damage to DNA [21,76,77]. Radiation in the 260–270 nm range is strongly absorbed by nucleic acids, inducing the formation of photoproducts, mainly cyclobutane pyrimidine dimers (CPDs) and 6-4 photoproducts (6-4PPs) [21,78]. These lesions distort the DNA helix, blocking replication and transcription, ultimately leading to cell death [21]. According to [23], DNA damage induced by UV-LED treatment (maximum at 266 nm) in *S. pastorianus* and *Pichia membranaefaciens* is the primary factor associated with yeast inactivation. However, significant membrane damage was also observed at 279 nm, attributed to protein absorption. This is due to the amino acids tyrosine, tryptophan, and phenylalanine. Of these, tryptophan has a maximum absorption peak around 280 nm. The main proteins that are altered are transporters and permeases, causing an osmotic imbalance and a lack of nutrients. Enzymes responsible for maintaining the proton gradient, such as membrane ATPase, may also be affected. Flow cytometry and transmission electron microscopy (TEM) studies in *S. cerevisiae* have demonstrated that UV-C radiation causes loss of cytoplasmic membrane integrity (permeabilization) and decreased intracellular enzymatic activity (esterase activity), revealing substantial intracellular disorganization, with coagulation of internal contents and detachment of the cell wall [62,79]. Broad spectrum light pulses (200–1100 nm), employs a multi-mechanism inactivation process that includes photochemical (UV), photothermal (overheating), and photophysical effects [54]. A study on *S. cerevisiae* demonstrated that high peak power of PL induces severe membrane damage, evidenced by increased protein leakage and visible structural alterations (vacuole expansion and membrane distortion), effects not observed under continuous UV-C exposure [55].

Species variability is a critical factor. In UV-C studies, the genus *Saccharomyces* (e.g., *S. cerevisiae*) has shown the highest resistance, followed by *Zygosaccharomyces* (*Z. bailii*), while *Dekkera* (*B. bruxellensis*) was the most sensitive [80]. However, when using PL-UV, *B. bruxellensis* proved to be highly sensitive (>6 log reduction in red wine; >4.7 log in white wine), whereas yeasts such as *Metschnikowia pulcherrima*, *Starmerella bacillaris*, and *Lachancea thermotolerans* exhibited extreme resistance [54,68]. This discrepancy highlights the distinct mechanisms of action; the resistance of vineyard yeasts (e.g., *M. pulcherrima*) to PL-UV may represent an ecological adaptation to sunlight [54].

### 4.3. Critical Matrix Factors and Processing Improvements Strategies

The effectiveness of yeast inactivation is primarily limited by the UV source, the optical properties of the food matrix and the dose applied as shown in Table 1. High UV absorbance, defined as the capacity of a matrix to absorb UV radiation, denoted by the absorption coefficient (α; cm^−1^), provides UV absorption values at a specific wavelength as a function of the optical path length penetrated by the material [20,37]. This parameter is influenced by components such as suspended solids, pulp, sugars, proteins, and pigments (particularly anthocyanins), which absorb and scatter light, preventing it from penetrating the liquid and reaching microorganism’s DNA [81]. Three phenomena occur when UV light interacts with solids. First, absorption, where the component sequesters UV energy, generally given by aromatic amino acids such as tryptophan, tyrosine, and phenylalanine. Second, the scattering of UV rays in different directions, reducing the intensity with which they penetrate the fluid. And finally, reflection where UV light reflects off the matrix. Components such as fiber in the case of fruit juices can hide the yeast, preventing UV energy from penetrating the microorganism [81]. Ref. [82] demonstrated that UV-C inactivation of spoilage yeasts is strongly dependent on matrix optical properties. At doses up to 1720 mJ cm^−2^, *S. cerevisiae* showed reductions of 3.2 log in melon juice, decreasing to 2.7 log in carrot juice and 1.7 log in orange juice, while *Zygosaccharomyces bailii* exhibited approximately 2.0 log reduction in orange juice. In clear grape juice, a 3.39 log reduction in *S. cerevisiae* was achieved at 65.5 mJ cm^−2^, whereas in turbid grape juice only 1.54 log reductions were obtained at 78.56 mJ cm^−2^, evidencing reduced UV penetration in the presence of suspended particles [83]. Similarly, Ref. [84] systematically compared turbid and clarified apple and grape juices and showed that in apple juice, the dose required for the first log reduction increased from 1485 J L^−1^ in clarified juice (≈53 NTU) to 1902 J L^−1^ in turbid juice (≈2100 NTU). As turbidity increases, substantially higher UV energy inputs are required to overcome absorption and shielding effects, although clarified or low-turbidity juices require comparatively lower energy densities to achieve similar microbial reductions. To overcome poor penetration in opaque liquids, reactors generating secondary turbulent flows have been developed to improve microbial exposure to UV light [85].

Yeast inactivation curves under UV-C often exhibit an initial adaptation phase followed by log-linear kinetics [69,86]. This adaptation indicates that the cell can repair sublethal UV-induced damage until a critical threshold is surpassed [80]. This phenomenon may lead to viable but non-culturable cells (VBNCs), which retain metabolic activity and pose a risk of recovery [69,79]. The persistence of this VBNC state in food matrices is a critical concern, as these cells can remain metabolically active and potentially pathogenic or spoilage capable, depending on different factors such as temperature or nutrients availability [69,79].

In beverage systems stored under refrigeration, microbiological stability has been reported for 12–30 days in fruit juices and up to 12 weeks in wine treated with UV-C, suggesting that long-term regrowth is unlikely when products are maintained under dark, cold storage [87,88,89,90]. However, if the food product is subsequently exposed to visible light, the mechanism of photoreactivation can be triggered. This enzymatic repair process, mediated by photolyase, can occur within the first hours after irradiation (typically within 4–24 h) reversing UV-induced DNA damage and potentially transitioning cells from a dormant VBNC state back to an actively proliferating state. After this early window, the probability of recovery decreases progressively, particularly under refrigeration and in the absence of light. Combining UV-C with mild heat has shown a strong synergistic effect, eliminating the initial adaptation phase of the inactivation curve and effectively removing VBNCs [79,80,91]. Likewise, combining Pulsed Light (PL) with natural antimicrobials (e.g., nisin and polylysine) has proven more effective for inactivating *S. cerevisiae* than PL alone [70].

**Table 1:** Applications of UV-C Lamps, UV-LED, and Pulsed Light in the Inactivation of Yeasts in Different Matrices.

UV Technology Type	Wavelength (nm)	Matrix	Yeast Species	Dose/Fluence	Log Reduction	Observations	Reference
LP-UV	254	Commercial orange juice	17 spoilage yeast strains (*Candida*, *Pichia*, *S. cerevisiae*, *Torulaspora*, etc.)	*Candida parapsilosis* = 245 mJ cm^−2^; *Cryptococcus albidus* = 1924–2175 mJ cm^−2^	1 log	Variable resistance: *Cryptococcus albidus* > *S. cerevisiae* > *C. parapsilosis*	[92]
LP-UV	254 nm	Fresh-cut apple	*Candida sake*, *H. uvarum*, *P. fermentans*, *M. pulcherrima*	2.5–10 kJ m^−2^ (≈ 250–1000 mJ cm^−2^)	1.72–1.81 log	7.5–10 kJ m^−2^ showed the highest yeast reduction and limited regrowth during refrigerated storage	[93]
LP-UV	254	Model wine/real wine	*S. cerevisiae* D576	200–1500 J L^−1^	>5 log	Influence of flow type (laminar, turbulent, Dean); FEP-coiled reactor most efficient	[94]
LP-UV	254	Red wine/solid media	*Brettanomyces bruxellensis*, *S. cerevisiae*, *L. thermotolerans*	0–10,000 µJ cm^−2^; liquid red wine: up to 6624 J L^−1^	5-log reduction for *B. bruxellensis*; lower sensitivity for *S. cerevisiae*	Marked inter-/intra-variability; *Brett.* more sensitive; inactivation at 6624 J L^−1^ in red wine; CPD lesions confirmed	[95]
LP-UV	253.7	Cabernet Sauvignon red wine	*Brettanomyces bruxellensis* (6 strains)	6624 J L^−1^	5 out of 6 strains achieved 5 log reduction	Genetic-group dependent sensitivity; wine’s high turbidity reduced efficacy; UV-C induces CPD-mediated DNA damage	[95]
LP-UV	254	Clarified and turbid white grape juice	*S. cerevisiae*	900 mJ cm^−2^	Turbid juice: 4.36 log; Clarified: 5 log	UV-C achieved 4.36 log reduction in *S. cerevisiae* at 900 mJ cm^−2^ in 20 min	[96]
LP-UV + heat (50 °C)	254	Pear, orange-tangerine, and tropical blend juices	*S. cerevisiae*	390 mJ cm^−2^	4.4–5.5 log	UV-C/Heat synergy; no recovery during storage	[91]
LP-UV + encapsulated citral/vanillin	254	Orange Tangerine and orange-banana-mango kiwi-strawberry juices	*S. cerevisiae*	390 mJ cm^−2^	1.5–1.6 log	Membrane damage and presence of sublethal cells	[97]
LP-UV + heat (50 °C)	254	Apple + raspberry juice	yeasts and molds	9.68 mJ cm^−2^	Significant initial reduction	UV-C alone induced a significant reduction in yeasts and molds while combined treatment showed no growth	[98]
Pulsed Light (PL)	200–1100 (25% UV-C)	Red wine/solid media	*Brettanomyces bruxellensis*, *S. cerevisiae*, *L. thermotolerans*	5–22.8 J cm^−2^	>6 log for *B. bruxellensis*; 2–4 log for other wine yeasts	Photochemical + photothermal effects; membrane and vacuolar disruption; high inter-/intra-specific variability	[54]
Pulsed Light (PL) + mild heat	200–1100	Verjuice (green grape juice)	*S. cerevisiae* NRRL Y-139	6–34 J cm^−2^ + 45–47 °C	5.0 log	PL + MH at 47 °C >5 log; minimal optical alteration; 6-week storage without yeast recovery	[99]
UV-LED	279	Orange juice	*S. cerevisiae*	160–1420 mJ cm^−2^	Up to 4.44 log	Severe membrane damage; DNA/protein leakage; SEM shows cell collapse	[100]
UV-LED	275	Apple juice	*Zygosaccharomyces rouxii*	800–1200 mJ cm^−2^	4.86 log at 800 mJ cm^−2^; 5.46 log at 1200 mJ cm^−2^	Membrane damage and loss of integrity (PI)	[101]
UV-LED	266–279	Solid culture media	*S. pastorianus*, *P. membranaefaciens*	0.1–0.6 mJ cm^−2^	1–4 log	Increased permeability (PI, DiBAC_4_(3)); greater DNA damage at 266 nm → CPDs; secondary membrane damage	[23]
UV-LED	280 and 365	Clear and turbid apple juice	*S. cerevisiae*	707–1100 mJ cm^−2^	1.6–4.4 log	UV-C LED (280 nm) more effective than UV-A; efficacy reduced in turbid juice	[86]

Resistance of spoilage yeasts to UV treatment can also be described through decimal reduction parameters (D_10_), defined as the irradiation time or UV dose required to achieve a 1 log (90%) reduction in the microbial population under specific experimental conditions. When expressed in dose units, the parameter is referred to as DUV, which represents the UV-C energy (mJ/cm^2^ or J/mL) required to produce the 1 log reduction and corresponds to the product of irradiance and exposure time. In clear apple juice, UV-C D_10_ values ranged from 6.38 min for *Saccharomyces cerevisiae* to 11.04 min for *Pichia fermentans*, with composite inocula showing intermediate resistance (8.28 min), evidencing a high UV tolerance of yeasts in comparison to bacterial contaminants under similar conditions [102]. In coconut liquid endosperm, D values at 254 nm were markedly shorter in temporal terms (22.76–26.74 s), yet the corresponding DUV values ranged from 99.96 to 122.72 mJ/cm^2^ and reached 214.89 mJ/cm^2^ when lag phases were incorporated into D calculations [80,103]. Furthermore, Ref. [80] expressed microbial resistance as 4D values (dose required for a 4-log reduction), where *S. cerevisiae* required 3.92 J/mL to achieve a 4-log reduction, approximately 2.4-fold higher than the most sensitive *Dekkera* spp., evidencing variability in UV tolerance among yeast species.

## 5. Yeast Repair Response

The existence of cellular mechanisms to repair UV-induced genetic damage has been recognized for decades [104,105,106,107]. In yeasts, unicellular eukaryotic organisms such as *Saccharomyces cerevisiae*, these repair systems are essential for post-irradiation cell survival. The primary genotoxic lesion caused by UV-C radiation (200–280 nm) is the formation of pyrimidine dimers (CPDs) and, to a lesser extent, pyrimidine photoproducts (6-4 photoproducts, 6-4PPs) [21,104,105,108].

Yeast cells have genetically evolved various pathways to address this damage such as Photoreactivation (PR), Nucleotide Excision Repair (NER), and Recombinational (or post-replicative) Repair (HR) [21,78,109,110]. The presence and efficiency of these pathways determine the shape of inactivation curves; the appearance of an initial shoulder in yeast dose–response curves is directly attributed to the ability of these mechanisms to repair non-lethal damage before it accumulates and becomes lethal [69,79,80].

### 5.1. Photoreactivation (PR)

Photoreactivation (PR), also known as photorepair, is a light-driven enzymatic repair mechanism and the most direct system for reversing pyrimidine dimers (CPDs) formed by UV radiation [78,105,110,111]. This mechanism relies on a single enzyme, photolyase which binds specifically to CPDs in DNA and, using light energy in the 300–500 nm range (visible and UV-A light), catalyzes the monomerization (splitting) of the dimer, restoring the two original nucleotides [112,113].

In *Saccharomyces cerevisiae*, photolyase is encoded by the *PHR1* gene [78,109,110]. The importance of this gene is absolute for this mechanism; studies in PHR1 mutants have demonstrated that yeast photoreactivation capacity is almost completely abolished in the absence of this enzyme [78,110]. This mechanism has also been identified as a key repair pathway in other yeasts of clinical and food relevance, such as *Candida albicans* [114]. Although highly efficient, this pathway is entirely dependent on subsequent exposure to visible light and cannot occur in darkness [111,115].

### 5.2. Nucleotide Excision Repair (NER)

NER, often referred to as dark repair, is the main light-independent pathway in yeasts for removing pyrimidine dimers and other types of lesions that distort the DNA helix [37,105,110]. Unlike photoreactivation, NER is a complex process involving multiple proteins [78]). The mechanism recognizes the structural dis) and caused by the dimer, cleaves the DNA strand on both sides of the lesion, removes an oligonucleotide containing the damage (approximately 24–32 nucleotides), and finally resynthesizes the correct sequence using the intact complementary strand as a template [105,116].

In *S. cerevisiae*, this pathway is controlled by the RAD (Radiation sensitive) gene group [109,110]. The genes RAD1, RAD2, RAD3, RAD4, RAD7, RAD10, RAD16, and RAD23 are essential components of the NER machinery [104,110,116,117]. The RAD3 gene is vital, as its mutations render yeast extremely sensitive to UV radiation and impair the incision step of DNA repair. Similarly, strains lacking RAD1 or RAD2 exhibit extreme UV sensitivity and have almost no excision repair capacity [104,116]. UV-B exposure also actively induces the expression of several of these RAD genes (including RAD2, RAD7, RAD16, and RAD23), indicating that yeast responds to genetic damage by increasing its repair capacity [117].

### 5.3. Recombinational Repair

When DNA lesions (such as dimers) are not removed by NER or PR before the cell attempts to replicate its genome, the replication machinery (DNA polymerase) stalls upon encountering the dimer. This results in a gap or a double-strand break (DSB) [105,118]. The recombinational repair pathway, also known as post-replicative repair (PRR), is activated to repair these gaps [78,109,110].

This mechanism uses homologous recombination to repair the damage [21]. Instead of removing the dimer, the cell uses the sister chromatid as a template to fill the gap in the damaged strand, effectively bypassing the lesion [105,118]. The original dimer remains in the parental strand, but genome integrity is restored, allowing replication to continue. In S. *cerevisiae*, this pathway critically depends on the RAD52 gene group (which includes RAD51, RAD52, RAD54, among others) [78,109,110]. Mutants in these genes, although viable, are highly sensitive to UV radiation, confirming the essential role of recombination in tolerance to UV-induced damage [109,118].

### 5.4. Global Cellular Response and Mutagenesis

Exposure to UV radiation triggers a complex cellular response that extends beyond simple repair. UV radiation is a potent mutagenic agent [116]. Mutagenesis occurs when repair systems, particularly post-replicative pathways, fail to recognize the lesion and replication proceeds despite the damage, a process known as translesion synthesis (TLS) [105].

The NER pathway (specifically the RAD1 gene) plays a crucial role not only in survival but also in preventing mutagenesis. RAD1 mutants exhibit significantly higher UV-induced mutation rates compared to wild-type strains [116]. This suggests that when the precise NER pathway fails, DNA lesions are channeled into damage-tolerance pathways (such as TLS) that are inherently more error-prone, resulting in mutations [116]. In addition to the RAD group, UV-B exposure also induces the expression of other key genes involved in DNA replication and repair, such as CDC9 (encoding DNA ligase I) and RPB2 (a subunit of RNA polymerase II), demonstrating a coordinated and integrated cellular response to genotoxic stress [117].

## 6. UV-Induced Yeast Mutagenesis in Bioprocesses

UV radiation has long been recognized as an effective physical mutagen capable of generating heritable genetic variability in *Saccharomyces cerevisiae* and other industrially relevant yeasts. UV exposure induces DNA lesions, primarily CPDs and 6-4 photoproducts, which, when not correctly repaired, can be converted into stable mutations through DNA damage tolerance pathways, particularly translesion synthesis (TLS) [116,119,120,121].

UV-induced mutagenesis in yeast is a regulated outcome of DNA damage processing, determined by the balance between error-free repair (NER, BER) and error-prone pathways involving Rev1 and DNA polymerase ζ (Polζ) [122,123,124,125]. This interaction between cell survival and genomic stability underlies the use of UV irradiation as a tool to generate phenotypic diversity relevant to bioprocesses.

From a bioprocess perspective, the genetic variability generated by UV irradiation provides the possibility for improvement of frequently used yeasts in the food industry. Because UV-induced mutations frequently affect regulatory pathways controlling stress response, membrane composition and redox balance, this approach enables the emergence of yeast variants with altered fermentation performance, tolerance to inhibitory compounds, or modified metabolite production profiles. The phenotypic outcome is not dictated by the irradiation itself, but rather by subsequent screening and selection under defined process-related pressures, which allow the enrichment of strains displaying improved robustness or functional characteristics.

Following this approach, UV-induced mutagenesis constitutes a non-recombinant strategy for generating phenotypic diversity, as it does not rely on in vitro nucleic acid technologies or the deliberate insertion of recombinant or synthetic DNA sequences that characterize genetically engineered microorganisms (GEMs) in modern food biotechnology [126]. Within the European Union framework, organisms whose genetic material has been altered beyond natural mating or recombination are generally considered genetically modified [127]. Nevertheless, the Court of Justice of the European Union clarified that only conventional mutagenesis techniques with a long history of safe use remain excluded from the main genetically modified organisms (GMO) regulatory obligations, whereas newly developed targeted mutagenesis approaches do not automatically qualify for this exemption. In the context of microorganisms, regulatory classification has direct implications for authorization procedures, labeling considerations, and market access. Accordingly, classical UV mutagenesis has historically been distinguished from recombinant genetic engineering, although its regulatory status ultimately depends on its recognition as a conventional technique under the applicable framework.

### 6.1. Functional Consequences of UV-Induced Mutagenesis in Yeasts

Several studies have established that UV-induced mutations are fixed primarily during DNA replication, when unrepaired lesions are bypassed by TLS polymerases [116,120,121]. Polη generally promotes relatively accurate bypass of CPDs, whereas Rev1 and Polζ introduce substitutions, frameshifts and clustered mutations, generating broad genetic diversity [123,124].

Recent work has expanded this view by demonstrating that UV exposure can also induce large-scale genomic alterations, including mitotic recombination and loss of heterozygosity, particularly when irradiation occurs outside of S phase. Ref. [128] showed that UV irradiation of G1-arrested diploid yeast induces recombination events at frequencies exceeding those of point mutations, revealing an additional layer of UV-driven genomic diversification. Such structural variations can profoundly affect gene dosage, regulatory networks and metabolic fluxes, with direct implications for strain performance in bioprocesses.

In parallel, studies focusing on different regions of the UV spectrum have demonstrated that mutational outcomes depend strongly on lesion type and repair context. While UVB/UVC predominantly induces helix-distorting lesions processed by NER and TLS, UVA promotes oxidative DNA damage, particularly 8-oxoguanine, whose mutagenic potential is revealed when base excision repair is limiting [108,129]. These mechanistic distinctions suggest that wavelength, dose and cellular context can be strategically modulated to shape the mutational landscape generated by UV treatment.

### 6.2. UV-Induced Yeast Improvement for Bioprocess Applications

Various authors in this topic demonstrated that UV-induced mutagenesis can yield yeast strains with superior performance under industrially relevant conditions. Several studies have reported significant improvements in ethanol tolerance, thermotolerance and osmotolerance following UV mutagenesis, translating into higher ethanol yields, productivities and robustness under high-gravity or high-temperature fermentations [130,131,132,133].

For instance, Ref. [130] obtained UV-derived mutants of thermotolerant *Saccharomyces cerevisiae* capable of sustained growth and ethanol production at 42 °C, whereas the parental strain showed a strong decline in performance above 37 °C. In a similar context, Ref. [133], treated *Saccharomyces cerevisiae* with a UV wavelength of 234 nm and reported a UV-mutant strain that tolerated up to 15% (*v*/*v*) ethanol and produced approximately 10.3% (*v*/*v*) ethanol from molasses, corresponding to a productivity of 1.7 g L^−1^ h^−1^ and a yield close to 99% of the theoretical maximum, clearly outperforming the wild-type strain under ethanol-stress conditions.

More pronounced results were reported by [132], who showed that UV mutagenesis outperformed chemical mutagenesis in generating high-performing strains of *S. cerevisiae*. The selected UV mutant reached ethanol concentrations of 122.2 ± 2.8 g L^−1^ at 37 °C, while maintaining tolerance to 21% (*v*/*v*) ethanol and 50% (*w*/*v*) glucose, values that were not achieved by the parental strain or by chemically mutagenized variants. Ref [111] reported UV-derived mutants capable of producing 41–42 g L^−1^ ethanol during simultaneous saccharification and fermentation at 40 °C, representing an increase of approximately 8–9 g L^−1^ compared with the parental strain under the same conditions. Similarly, Ref. [134] reported that *Saccharomyces cerevisiae* strains exposed to UV-C irradiation (254 nm) increased ethanol yields by 35–45%, reaching approximately 8.5% (*v*/*v*) ethanol compared with 6.0–6.5% (*v*/*v*) in the non-irradiated strains.

UV mutagenesis has also proven effective in non-conventional yeasts relevant to food and beverage bioprocesses. Ref. [135] reported that a UV-mutagenized strain (254 nm) of *Wickerhamomyces anomalus* increased glycerol production from 66.6 to 80.2 g L^−1^ while also exhibiting tolerance to 30% (*v*/*v*) ethanol and 4 M NaCl. Similarly, Ref. [136] showed that UV-derived mutants of *Pichia terricola* increased ethanol tolerance from 8% to 10–11% (*v*/*v*) and altered the production of volatile esters during wine fermentation, demonstrating that UV-induced mutations can simultaneously affect stress tolerance and metabolic profiles. Ref. [137] demonstrated that UV-induced mutagenesis, using UV-C irradiation at 234 nm, obtained *Scheffersomyces stipitis* mutants capable of growing under anaerobic conditions on xylose, a phenotype absent in the parental strain. Selected mutant strains produced up to 13 g L^−1^ ethanol under strictly anaerobic conditions. These studies highlight the potential of UV-induced mutagenesis as a strategy for quantitatively improving complex, polygenic traits across diverse yeast species used in bioprocess applications.

### 6.3. Opportunities and Limitations of UV-Based Approaches in Bioprocess Applications

From an industrial perspective, the main appeal of UV-based approaches, including UV-induced mutagenesis, lies in their operational simplicity, low implementation cost and broad regulatory acceptance, particularly in regions where the use of genetically modified organisms is limited or restricted. UV treatments can be readily integrated into existing process lines, require relatively simple equipment, and allow rapid generation of phenotypic diversity without the need for recombinant DNA techniques. These features make UV irradiation an attractive tool for strain improvement, especially when targeting complex, multigenic traits such as stress tolerance, metabolic robustness or adaptation to harsh process conditions.

However, the same biological mechanisms that enable UV to generate beneficial genetic diversity also impose important limitations. UV-induced DNA damage, if excessive or poorly controlled, can alter cellular repair systems and result in lethal effects, impaired growth, or unfavorable metabolic alterations, reducing process performance. Studies using DNA repair-deficient yeast strains have shown that high UV doses can lead to severe metabolic disruption and loss of viability, highlighting the need for sublethal irradiation treatments and conditions [138,139]. In addition to these immediate physiological effects, the long-term genetic stability of UV-derived mutants represents a critical consideration, particularly in continuous bioreactor systems. UV irradiation primarily induces point mutations and small genomic alterations processed through translesion synthesis pathways involving Polη and Rev1/Polζ [120,125]. Once fixed in chromosomal DNA, such mutations are generally stable and unlikely to revert spontaneously to wild-type. Nevertheless, phenotypic instability may emerge during continuous cultivation due to compensatory mutations or selective pressures inherent to industrial processes. Therefore, beyond initial strain selection, UV mutagenesis strategies should incorporate generational stability assessments to ensure that improved traits remain genetically fixed and robust under continuous operational conditions. Consequently, UV mutagenesis must be coupled with effective selection procedures to distinguish stable, high-performing variants from transient or detrimental phenotypes.

An additional limitation arises from the genomic complexity of UV-induced changes. Beyond mutations, UV exposure can promote large-scale genetic events, including mitotic recombination and loss of heterozygosity, particularly under specific cell cycle conditions [128]. While such events can accelerate phenotypic diversification, they may also compromise long-term genetic and phenotypic stability, which is a critical requirement for industrial bioprocesses operating under continuous or repeated batch modes. Instability can lead to performance drift over time, reducing process predictability and reproducibility.

Therefore, the effective industrial application of UV-based approaches requires more than simple irradiation. It demands optimization of irradiation parameters (dose, wavelength, exposure time), integration with rational selection and screening strategies, and systematic evaluation of phenotypic stability across multiple cultivation cycles. Given the high precision offered by modern UV-LEDs, there is a significant opportunity to explore synergistic effects through the combination of different wavelengths. This multi-wavelength approach could emerge as a viable strategy for fine-tuning functional modulation, allowing for more targeted physiological response compared to traditional methods. This already occurs in the case of bacterial inactivation where wavelength combinations are primarily employed to maximize inactivation efficiency and suppress repair mechanisms. Simultaneous UVA and UVC irradiation has produced synergistic bactericidal effects associated with delayed CPD repair and interference with mediated DNA repair pathways, combinations such as 280/300 nm have enhanced inactivation kinetics in *Campylobacter jejuni*, and polychromatic emission from medium-pressure UV lamps has reduced photoreactivation in *Escherichia coli*, likely through the concurrent induction of multiple DNA lesions and impairment of photolyase-mediated repair [112,140,141]. Comprehensive reviews further indicate that combining UVA, UVB, and UVC can enhance microbial inactivation and limit cellular reactivation in bacterial models [142]. However, these multi-wavelength strategies have been investigated almost exclusively for microbial inactivation and not for functional modulation or mutagenesis purposes. Moreover, direct extrapolation of synergistic effects from bacteria to *Saccharomyces* and non-*Saccharomyces* yeasts would be inappropriate due to fundamental structural and physiological differences, including structure organization, distinct DNA repair networks, and differences in oxidative stress responses. Consequently, while multi-wavelength UV-LED strategies represent a well-established tool for bacterial inactivation, their potential application for controlled yeast modulation remains unexplored and warrants systematic investigation. When these aspects are properly addressed, UV-based technologies can offer a practical and versatile platform for yeast improvement, but their success ultimately depends on balancing diversity generation with robustness and stability under real process conditions.

## 7. Conclusions and Future Outlook

Ultraviolet (UV) technology has become an important alternative for the disinfection of food-related surfaces and liquid matrices; however, in yeast-based applications, its potential extends beyond microbial inactivation. Depending on irradiation conditions, UV treatments can enable complete inactivation, regulation of yeast viability, or induce genetic changes that may influence functional and technological performance in bioprocesses. In this context, further research is required to fully exploit the potential of UV-LED systems, as they offer a higher degree of control over irradiation parameters, such as wavelength, intensity, and dose applied compared to conventional UV technologies. Future research must transition from conventional *Saccharomyces cerevisiae* models toward a systematic exploration of non-*Saccharomyces* and non-conventional yeasts. Given their distinct DNA repair mechanisms, stress responses, and metabolic traits, offering a broader chemical landscape for UV-induced modulation of fermentation and aroma profiles. To ensure industrial scalability, greater emphasis should be placed on mapping specific wavelength response curves to define lethal versus stimulatory thresholds, assessing the phenotypic stability under industrial conditions and combining UV treatments with screening to decode the metabolic shifts driving yeast modulation. Ultimately, shifting the paradigm of UV light from a simple antimicrobial tool to a precision bioprocess regulator will be essential for diversifying food and fermentation biotechnology.

## Data Availability

No new data were created or analyzed in this study. Data sharing is not applicable to this article.

## Abbreviations

AT	Adenine-Thymine
6–4PPs	Pyrimidine (6–4) pyrimidone photoproducts
BER	Base Excision Repair
CPDs	Cyclobutane Pyrimidine Dimers
DNA	Deoxyribonucleic Acid
DSB	Double-Strand Break
GC	Guanine–Citosine
GEM	Genetically modified microorganisms
GMO	Genetically modified organisms
HO	Hydroxyl radical
HR	Homologous Repair
LED	Light-Emitting Diode
LP-UV	Low-Pressure Ultraviolet
MH	Mild Heat
NER	Nucleotide Excision Repair
PL	Pulsed Light
PL-UV	Pulsed Ultraviolet Light
Polζ	DNA polymerase ζ
Polη	DNA polymerase η
PR	Photoreactivation
PRR	Post-Replicative Repair
ROS	Reactive Oxygen Species
TEM	Transmission Electron Microscopy
TLS	Translesion Synthesis
UV	Ultraviolet
UV-A	Ultraviolet A radiation
UV-B	Ultraviolet B radiation
UV-C	Ultraviolet C radiation
UV-LED	Ultraviolet Light-Emitting Diode
VBNC	Viable But Non-Culturable
VUV	Vacuum Ultraviolet
